# Incidence rate and topography of intra-pelvic arterial lesions associated with high-energy blunt pelvic ring injuries: a retrospective cohort study

**DOI:** 10.1186/s12873-021-00470-y

**Published:** 2021-06-30

**Authors:** Anna-Eliane Abboud, Sana Boudabbous, Elisabeth Andereggen, Michaël de Foy, Alexandre Ansorge, Axel Gamulin

**Affiliations:** 1 Division of Orthopaedic and Trauma Surgery, University Hospitals of Geneva, 4 Rue Gabrielle-Perret-Gentil, CH-1211 Geneva 14, Switzerland; 2 Department of Radiology, University Hospitals of Geneva, 4 Rue Gabrielle-Perret-Gentil, CH-1211 Geneva 14, Switzerland; 3 Division of Emergency Medicine, University Hospitals of Geneva, 4 Rue Gabrielle-Perret-Gentil, CH-1211 Geneva 14, Switzerland

**Keywords:** High-energy pelvic ring injury, High-energy blunt trauma, Intra-pelvic arterial lesion, Incidence, Topography

## Abstract

**Background:**

The aim of this study was to determine the rate and topography of intra-pelvic arterial lesions associated with high-energy blunt pelvic ring injuries (PRI).

**Methods:**

This retrospective cohort study was conducted in a level I trauma center serving 500,000 inhabitants. A total of 127 consecutive patients with high-energy blunt PRI were included between January 1st, 2014 and December 31st, 2017. Every patient had a total body or thoraco-abdominal computed tomography scan including contrast enhanced arterial sequences. A board-certified radiologist reviewed all the vascular images and precisely described every intra-pelvic arterial lesion in terms of localization. Complete pelvic series (standard radiographs and fine cut computed tomography images) were reviewed by three board-certified orthopedic surgeons experienced in PRI management, and Young and Burgess and AO/OTA classifications were determined. Demographic, clinical, therapeutic and outcome data were extracted from the institutional *severely injured patients’ registry*.

**Results:**

Patients’ mean age was 45.3 years and 58.3% were males. Fifteen (11.8%) had a total of 21 intra-pelvic arterial lesions: seven lesions of the obturator artery, four of the superior gluteal artery, three of the inferior gluteal artery, two of the vesical artery, and one of each of the following arteries: internal iliac, internal pudendal, fifth lumbar, lateral sacral, ilio-lumbar. These lesions occurred in 8.6% of lateral compression injuries, 33.3% of anteroposterior compression injuries and 23.5% of vertical shear and combined mechanism injuries (Young and Burgess classification, *p* = 0.003); and in 0% of type A injuries, 9.9% of type B injuries and 35% of type C injuries (AO/OTA classification, *p* = 0.001). Patients with an intra-pelvic arterial lesion were more likely to present with pre-hospital hemodynamic instability (*p* = 0.046) and to need packed red blood cells transfusion within the first 24 h (*p* = 0.023; they needed a mean of 7.53 units vs. 1.88, *p* = 0.0016); however, they did not have a worst outcome in terms of complications or mortality.

**Conclusions:**

This systematic study found an 11.8% rate of intra-pelvic arterial lesion related to high-energy blunt PRI. The obturator, superior gluteal and inferior gluteal arteries were most often injured. These findings are important for the aggressive management of high-energy blunt PRI.

**Supplementary Information:**

The online version contains supplementary material available at 10.1186/s12873-021-00470-y.

## Background

High-energy blunt pelvic ring injuries (PRI) represent a heavy burden for any institution taking care of severely injured patients [1–3]. Their incidence ranges from 1 to 10 cases/100′000/year [4–6].

Hemodynamic instability occurs in up to 17% of high-energy blunt PRI [7, 8], but an objective and specific rate is difficult to determine due to a lack of consensus in the definition of hemodynamic instability and the uncertainty about possible inclusion of low-energy PRI in some studies. Mortality rate in hemodynamically unstable PRI patients may be as high as 32% [7–10], with hemorrhage being the most common cause of death [8, 11, 12]. Hemodynamic instability may be caused by an intra-pelvic or extra-pelvic vascular injury [13]. Intra-pelvic bleeding occurs in almost all cases from bone fracture lines and venous lacerations, and in 10–20% of the cases from an arterial tear [12–18]. Osseous and venous bleeding related to PRI is usually controlled by mechanical stabilization of the pelvis sometimes associated with pelvic packing [13, 19]. Arterial bleeding associated with PRI may need additional angiography and embolization or resuscitative endovascular balloon occlusion of the aorta (REBOA) for optimal management [13, 14, 19, 20].

Publications on arterial injuries related to PRI are scarce and for some of them date back to more than 10 years ago [16, 17, 21–28]. The aim of the present study was therefore to determine the current rate of intra-pelvic arterial lesions (IPAL) associated with high-energy blunt PRI, as well as their topography, in order to update knowledge on these lethal injuries and optimize their aggressive management.

## Methods

### Study population and design

The setting of this retrospective cohort study was a 1900-bed urban academic medical center serving roughly 500,000 inhabitants (primary to tertiary care). This center is a level I trauma center according to the definitions of the Committee on Trauma of the American College of Surgeons and of the institution’s national medical authority [29, 30].

**Fig. 1:**
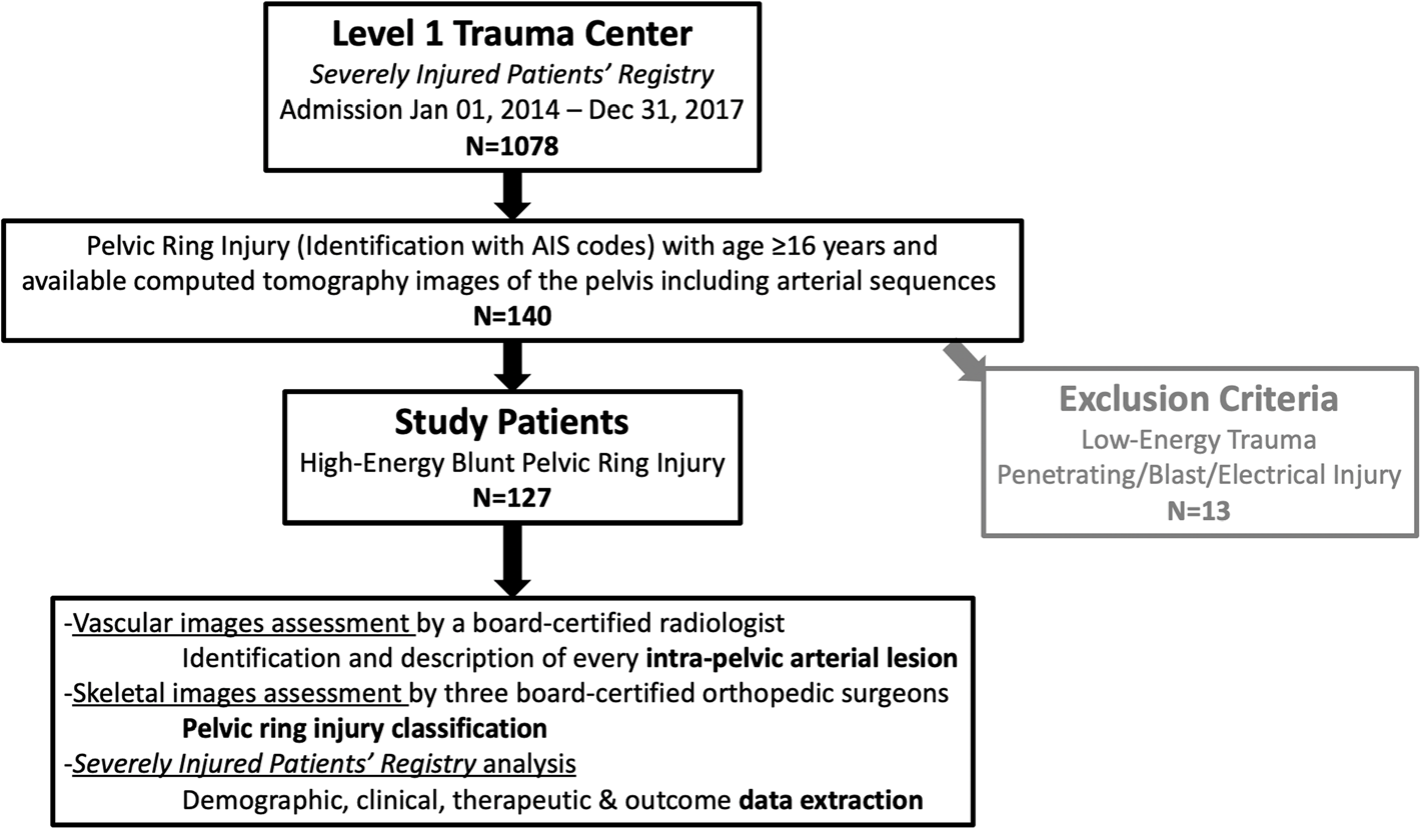
Study flowchart. This figure is authors’ own work

Identification of all consecutive patients admitted with a high-energy blunt pelvic ring injury was performed using the prospectively filled institutional *severely injured patients’ registry*. Among other items, Abbreviated Injury Scale (AIS) codes are reported in this registry [31]. Inclusion criteria were: 1) hospital admission between January 1st, 2014 and December 31st, 2017; 2) AIS code corresponding to a PRI (856,100.2, 856,101.3, 856,151.2, 856,152.3, 856,161.3, 856,162.4, 856,163.4, 856,164.5, 856,171.4, 858,172.4, 856,173.5, 856,174.5); 3) age ≥ 16 years old at the time of admission; and 4) available computed tomography (CT) images of the pelvis including arterial sequences. Exclusion criteria were: 1) penetrating trauma, blast injuries, electrical injuries; 2) and low-energy trauma (fall from patient’s own height). This left 127 patients with high-energy blunt PRI for the final analysis (Fig. 1).

### Outcome

The outcome was the presence of an IPAL related to the PRI. Every patient had a total body or thoraco-abdominal CT scan including contrast enhanced arterial sequences as part of the emergency management. Arterial sequences were obtained after intra-venous injection of 120 ml of Accupaque™ 350 (Iohexol, GE Healthcare AG, Opfikon, Switzerland) diluted with sodium chloride. A region of interest (ROI) was placed on the aorta to exactly define the arterial phase, which was followed by venous series in all cases, and sometimes by late acquisitions if deemed necessary. Some patients additionally had angiography and embolization if needed. All the vascular images were reviewed by one of the authors (SB, board-certified radiologist), and every IPAL was noted and precisely described in terms of localization.

### Variables of interest

Demographic (age and sex), clinical (pre-hospital systolic blood pressure and heart rate), therapeutic (total number of packed red blood cells (PRBC) transfused during the first 24 h) and outcome data (survival or death, timing of death, length of stay in the intensive care unit, length of total hospitalization excluding rehabilitation, and presence of complication) were extracted from the *severely injured patients’ registry*, as well as AIS codes and Injury Severity Scores (ISS) [32, 33]. Definition of a complication was any serious condition developing after hospital admission and potentially aggravating the outcome (surgical wound complication, compartment syndrome, infection, pressure sore, thrombo-embolic event, organ failure, stroke, myocardial infarction, cardiopulmonary arrest). Definition of pre-hospital hemodynamic instability was: 1°) pre-hospital systolic blood pressure < 90 mmHg; or 2°) pre-hospital heart rate > 100 bpm. An accredited nurse calculated AIS codes and ISS [32, 33]. Complete pelvic image series (standard radiographs and fine cut CT images) were reviewed using an open-source picture archiving and communication system (PACS) workstation digital imaging and communications in medicine (DICOM) viewer (OsiriX, Pixmeo Sàrl, Bernex, Switzerland). Multiple plans two-dimensional and three-dimensional CT reconstructions were generated. Young and Burgess [21] and AO/OTA [34] classifications were consensually determined by three board-certified orthopedic surgeons with experience in PRI management (MdF, AA and AG).

### Statistical analysis

Continuous variables were described by their mean ± standard deviation (SD), median, and range, and categorical variables by their frequencies and relative percentages. We compared the mean number of PRBC transfused during the first 24 h by presence of IPAL at admission in the entire cohort by performing Mann-Whitney non-parametric test. The presence of IPAL was compared among categories of the Young and Burgess classification then among the AO/OTA classification by performing Fisher’s exact tests. We also compared the presence of IPAL among categories of thoracic, abdominal and head/neck injuries (> 2 versus ≤2) using either Chi-2 test or Fisher’s exact test, when expected frequencies were below 3.

In a second step, factors associated with pre-hospital hemodynamic instability on one hand and transfusion requirement during the first 24 h on the other hand were separately explored by univariate logistic regression analyses. Each association was reported using odds ratios (OR) with 95% confidence intervals (95% CI). In both models, the main predictor was the presence of an IPAL. We adjusted for the following confounders: gender and age in categories (16–39, 40–49, 50–69, and ≥ 70 years). We also tested for the Young and Burgess classification and ISS categorized as < and ≥ 25. We checked for the existence of multicollinearity (*collin* command using STATA) employing the variance inflation factor (VIF). If it was above 2, we considered that multicollinearity was problematic and simplified the multivariable models. We finally assessed the models’ adequacy by applying the Hosmer-Lemeshow’s test on final multivariable models.

In a third step, we explored the associations between factors and important outcome variables (death and complications) by performing logistic regression analyses, following the procedure used for pre-hospital hemodynamic instability.

All the analyses were performed using STATA IC 16.0 (Stata Corporation, College Station, TX, USA). Statistical significance was defined as *p* < 0.05.

## Results

There were 127 patients with high-energy blunt PRI included in the study. The mean age of the cohort was 45.3 ± 18.4 years (range 17.8–87.9) and 74 patients (58.3%) were male. Clinical, therapeutic and outcome data of the cohort are further described in Table 1.

**Table 1:** Description of patients included in the study (*n* = 127)

Variables	
Mean age at trauma (± SD, median, range), in years	45.3 (±18.4, 44.4, 17.8–87.9)
Male sex, n (%)	74 (58.3)
AO/OTA classification, n (%)	
A	26 (20.5)
B	81 (63.8)
C	20 (15.7)
Young and Burgess classification, n (%)	
Not classifiable	25 (19.7)
Lateral compression (LC1, LC2 & LC3)	70 (55.1)
Antero-posterior compression (APC1, APC2 & APC3)	15 (11.8)
Vertical shear & combined mechanism (VS & CM)	17 (13.4)
Mean ISS (± SD, median, range)	26.4 (±12.9, 22, 5–66)
ISS, n (%)	
< 16	22 (17.3)
≥ 16	105 (82.7)
< 25	68 (53.5)
≥ 25	59 (46.5)
Hemodynamic instability (prehospital systolic BP < 90 or HR > 100), n (%) (2 missing)	31 (24.8)
Transfusion within 24 h, n (%)	47 (37.0)
Mean units PRBC received within 24 h (± SD, median, range)	6.9 (±8.0, 4, 1–40)
IPAL, n (%)	15 (11.8)
Number of patients needing ICU stay, n (%)	75 (59.1)
Mean ICU stay (±SD, median, range), in days	10.6 (±12.6, 7, 0–73)
Mean hospital stay (±SD, median, range), in days	27.0 (±34.6, 15, 1–191)
Complications during hospital stay, n (%)	44 (34.6)
Intra-hospital death, n (%)	16 (12.6)
Mean time to death (±SD, median, range), in days	13.1 (±17.2, 4.5, 1–52)

*SD:* standard deviation; *AO/OTA:* Arbeitsgemeinschaft für Osteosynthesefragen / Orthopaedic Trauma Association; *ISS:* Injury Severity Score; *BP:* blood pressure in mmHg; *HR:* heart rate in bpm; *PRBC:* packed red blood cells; *IPAL:* intra-pelvic arterial lesion; *ICU:* intensive care unit

**Fig. 2:**
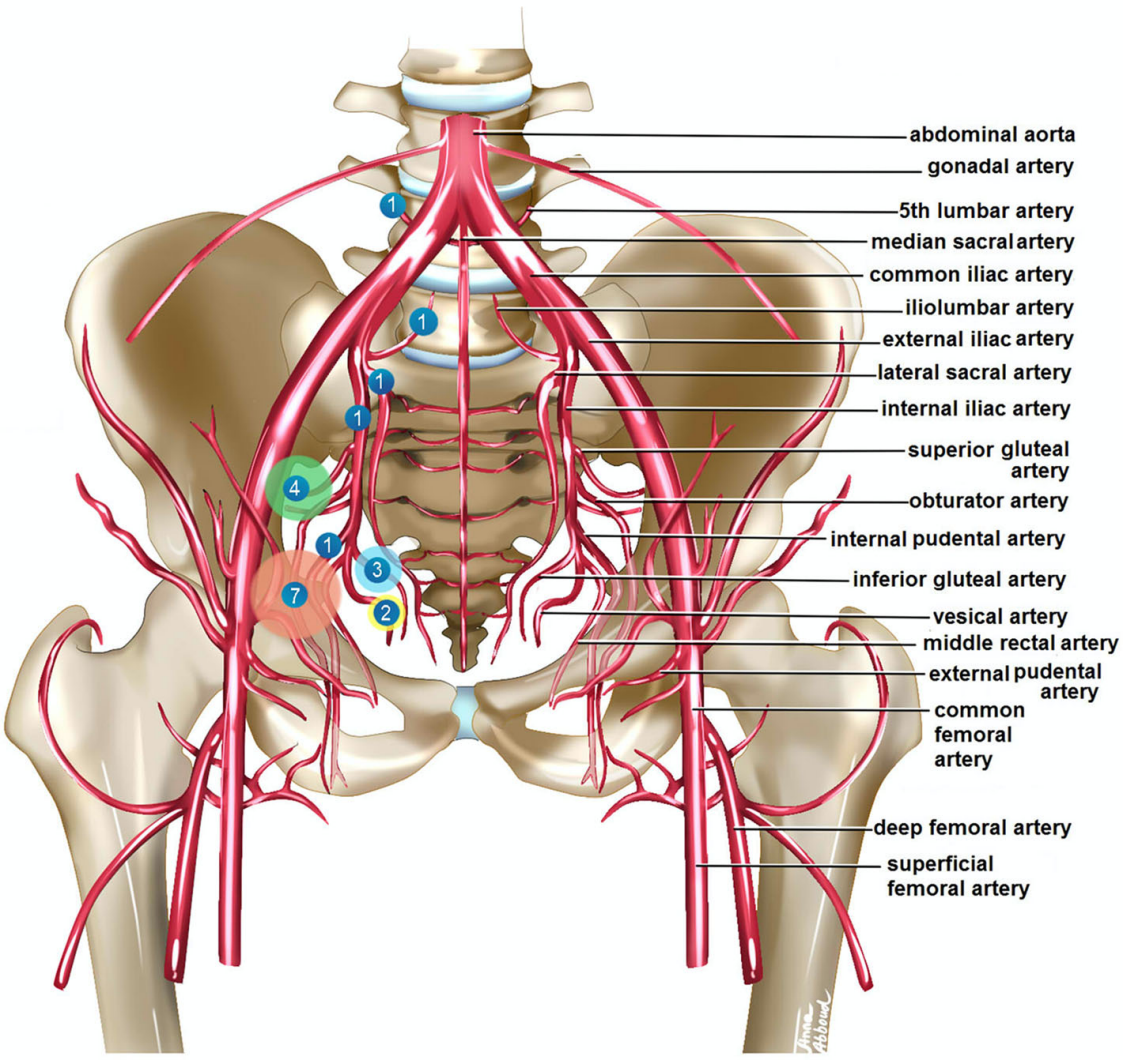
Topography of the intra-pelvic arterial lesions found in the study. All the lesions are arbitrarily reported on the right side of the pelvis for clarity purposes. Each circle corresponds to an arterial lesion location; the size of each circle is proportional to the number of arterial lesions found at the particular location, and the number within each circle refers to the number of cases found at the particular location. Artery names are reported on the left side of the pelvis. This figure is authors’ own work

Fifteen (11.8%) of the patients had one or more associated IPAL. A total of 21 IPAL were highlighted: seven lesions of the obturator artery, four of the superior gluteal artery, three of the inferior gluteal artery, two of the vesical artery, and one of each of the following arteries: internal iliac, internal pudendal, fifth lumbar, lateral sacral, ilio-lumbar (Table 2). Figure 2 shows the topography of the IPAL found in this study.

**Table 2:** Description of the 21 intra-pelvic arterial lesions found among 15 patients

Arteries	Patients with intra-pelvic arterial lesions (***n*** = 15)^**a**^	Selective embolization (***n*** = 6)^**b**^
Obturator artery	7 (46.7)	3 (42.9)
Superior gluteal artery	4 (26.7)	3 (75.0)
Inferior gluteal artery	3 (20.0)	2 (66.7)
Vesical artery	2 (13.3)	2 (100.0)
Internal iliac artery	1 (6.7)	0 (0.0)
Internal pudendal artery	1 (6.7)	0 (0.0)
Fifth lumbar artery	1 (6.7)	0 (0.0)
Lateral sacral artery	1 (6.7)	1 (100.0)
Ilio-lumbar artery	1 (6.7)	1 (100.0)

Values are expressed as n (%)

^a^ number of lesions found for each artery and rate of lesion among the 15 patients presenting an intra-pelvic arterial lesion

^b^ number and percentage of arterial lesions needing embolization

Six out of 15 IPAL patients (40.0%) needed selective embolization to control arterial bleeding and hemodynamic instability. Table 2 depicts the embolization sites. These six patients also needed PRBC transfusion during the first 24 h. Five other IPAL patients were managed with PRBC transfusion, and the remaining four did not need PRBC transfusion to control hemodynamic instability.

The presence of an IPAL was not associated with other severe extra-pelvic injuries (Supplement Table 1).

According to the Young and Burgess classification, IPAL occurred in 8.6% of lateral compression injuries, 33.3% of anteroposterior compression injuries and 23.5% of vertical shear and combined mechanism injuries (Table 3). These lesions were significantly more frequent in anteroposterior compression and vertical shear and combined mechanism type fractures (*p* = 0.003). According to the AO/OTA classification, IPAL occurred in 0.0% of type A injuries, 9.9% of type B injuries and 35.0% of type C injuries (Table 3). These lesions were significantly more frequent in type C injuries (*p* = 0.001).

**Table 3:** Association between intra-pelvic arterial lesions and type of fractures following the Young and Burgess and the AO/OTA classifications

	Arterial lesion		
Classification	Absent (***n*** = 112)	Present (n = 15)	***p***-value^**a**^
**Young and Burgess**			**0.003**
NC (*n* = 25)	25 (100.0)	0 (0.0)	
LC (*n* = 70)	64 (91.4)	6 (8.6)	
APC (*n* = 15)	10 (66.7)	5 (33.3)	
VS & CM (*n* = 17)	13 (76.5)	4 (23.5)	
**AO/OTA**			**0.001**
Type A (*n* = 26)	26 (100.0)	0 (0.0)	
Type B (*n* = 81)	73 (90.1)	8 (9.9)	
Type C (*n* = 20)	13 (65.0)	7 (35.0)	

Values are expressed as n (%), where % represents the percentage of fractures of each type associated with the presence or absence of an intra-pelvic arterial lesion

*NC:* not classifiable; *LC:* lateral compression; *APC:* anteroposterior compression; VS*:* vertical shear; *CM:* combined mechanism; *AO/OTA:* Arbeitsgemeinschaft für Osteosynthesefragen / Orthopaedic Trauma Association

^a^ Fischer’s exact test. Intra-pelvic arterial lesions were significantly more frequent for anteroposterior compression and vertical shear and combined mechanism type fractures; they were also more frequent for type type C fractures

Table 4 shows the analyses of factors associated with pre-hospital hemodynamic instability, and Table 5 of those associated with transfusion requirement during the first 24 h. Both pre-hospital hemodynamic instability and need for transfusion during the first 24 h were associated with IPAL in the multivariable analyses. The presence of an IPAL was associated with a higher mean number of PRBC transfused during the first 24 h (7.5 ± 11.5 (median 4, range 0–40) vs. 1.88 ± 4.33 (median 0, range 0–32); *p* = 0.0016).

**Table 4:** Factors associated with pre-hospital hemodynamic instability

	***Univariate analysis***			***Multivariable analysis***^***a***^		
Variables	OR	95% CI	***p***-value	OR	95% CI	***p***-value
IPAL (ref. no arterial lesion)	**6.00**	**1.93–18.64**	**0.002**	**4.48**	**1.03–19.54**	**0.046**
Female gender (ref. male)	1.27	0.56–2.88	0.569	1.67	0.60–4.61	0.325
Age in categories (ref. 16–39 years)			0.868			0.969
40–49	0.59	0.17–2.04	0.406	0.81	0.19–3.45	0.780
50–69	0.85	0.32–2.30	0.754	0.95	0.29–3.16	0.939
≥ 70 years	0.97	0.27–3.52	0.963	0.69	0.14–3.39	0.650
ISS, continuous	**1.13**	**1.08–1.19**	**< 0.001**	–	–	–
ISS ≥25 (ref. < 25)	**14.4**	**4.62–44.85**	**< 0.001**	**12.95**	**3.90–42.95**	**< 0.001**
AO/OTA classification (ref. A)			0.125	–	–	–
B	2.30	0.62–8.51	0.214			
C	4.67	1.04–21.01	0.045			
Young and Burgess classification (ref. NC)			0.267			0.948
LC	2.14	0.57–8.09	0.263	1.49	0.33–6.67	0.599
APC	2.42	0.46–12.85	0.298	1.76	0.20–15.59	0.611
VS & CM	4.67	0.99–22.01	0.052	1.60	0.26–9.87	0.612

After adjustment for sex and age, the presence of an IPAL was significantly associated with greater odds of pre-hospital hemodynamic instability independently of the types of PRI, as was ISS ≥25

*OR*: odds ratio; *95% CI*: 95% confidence interval; *IPAL:* intra-pelvic arterial lesion; *ISS:* Injury Severity Score; *NC:* not classifiable; *LC:* lateral compression; *APC:* anteroposterior compression; VS*:* vertical shear; *CM:* combined mechanism

^a^ Hosmer-Lemeshow test, *p* = 0.118. The variance inflation factors (VIF) are all below 1.5, suggesting no collinearity

**Table 5:** Factors associated with PRBC transfusion requirement during the first 24 h

	***Univariate analysis***			***Multivariable analysis***^***a***^		
Variables	OR	95% CI	***p-***value	OR	95% CI	***p***-value
IPAL (ref. no arterial lesion)	**5.81**	**1.73–19.49**	**0.004**	**4.53**	**1.23–16.66**	**0.023**
Hemodynamic instability (ref. none)	**28.50**	**8.85–91.74**	**< 0.001**	–	–	–
Female gender (ref. male)	0.69	0.33–1.45	0.331	0.81	0.37–1.79	0.610
Age in categories (ref. 16–39 years)			0.696			0.763
40–49	0.53	0.18–1.55	0.247	0.53	0.17–1.68	0.284
50–69	0.93	0.39–2.23	0.868	0.89	0.35–2.26	0.811
≥ 70 years	1.00	0.31–3.21	0.999	0.87	0.24–3.07	0.825
ISS, continuous	**1.17**	**1.11–1.24**	**< 0.001**	**–**	**–**	**–**
ISS ≥25 (ref. < 25)	**14.63**	**5.87–36.47**	**< 0.001**	**–**	**–**	**–**
AO/OTA classification (ref. A)			0.077	–	–	–
B	1.13	0.43–2.92	0.808			
C	3.38	0.99–11.46	0.051			
Young and Burgess classification (ref. NC)			0.367			0.688
LC	1.04	0.39–2.76	0.937	0.94	0.34–2.58	0.907
APC	1.86	0.50–6.94	0.356	1.31	0.30–5.77	0.723
VS & CM	2.39	0.67–8.51	0.179	1.87	0.48–7.24	0.365

^a^ Hosmer-Lemeshow test, *p* = 0.589. The variance inflation factors (VIF) are all below 1.2, suggesting no collinearity

After adjustment for sex and age, the presence of an IPAL was significantly associated with greater odds of PRBC transfusion independently of the types of PRI. Neither ISS nor hemodynamic instability was introduced in the regression model as both these factors were on the causal pathway from IPAL to transfusion

*OR*: odds ratio; *95% CI*: 95% confidence interval; *IPAL:* intra-pelvic arterial lesion; *ISS:* Injury Severity Score; *NC:* not classifiable; *LC:* lateral compression; *APC:* anteroposterior compression; VS*:* vertical shear; *CM:* combined mechanism

Further analysis did not highlight any significant association between the presence of an IPAL and complication (*p* = 0.260) or death (*p* = 0.206) occurrences. Complication occurrence was associated with severe thoracic injuries (*p* = 0.027), and death occurrence with severe head and neck injuries (*p* = 0.006).

## Discussion

This systematic study found an 11.8% rate of IPAL among patients sustaining high-energy blunt PRI. The three most often injured arteries were the obturator artery, the superior gluteal artery and the inferior gluteal artery. Present study results fall within the lower part of the range of IPAL reported in the literature (10–20%) [13–18]. These IPAL were diagnosed using contrast enhanced arterial sequences of emergency CT scans performed on all the patients of the study cohort as per local high-energy trauma patients’ management protocol. Only a small part of the cohort (10 patients) had additional angiography, raising the question of potential underdiagnosis of IPAL. Underdiagnosis did probably not happen, as CT scan with contrast enhanced arterial sequences is considered more sensitive than conventional angiography for detection of active arterial extravasation (0.3–0.5 mL/min bleeding rate detection for CT vs. 0.5–1.0 mL/min bleeding rate detection for conventional angiography) and also allows a rapid vascular cartography of the whole pelvis [35]. Performing an additional angiography on all the patients is controversial and time-consuming and might delay appropriate management of some multiply injured patients [13, 14, 35]. Therefore, CT scan imaging is validated as the first step management to provide adequate information regarding arterial bleeding (including IPAL) and potential need for additional angiography and embolization [13, 19, 23, 25, 35, 36].

A total of 21 IPAL were found among 15 patients sustaining high-energy blunt PRI. The obturator artery was most often injured (seven times out of 15 patients, 46.7%), followed by the superior gluteal artery (four times, 26.7%), the inferior gluteal artery (three times, 20.0%), the vesical artery (two times, 13.3%) and the internal iliac, internal pudendal, fifth lumbar, lateral sacral and ilio-lumbar arteries (each one time, 6.7%). The literature about arterial lesion topography in case of high-energy blunt PRI is scarce with only two recent retrospective studies [15, 27]. Metz et al. investigated on a series of 842 consecutive PRI patients, 49 of them having had a pelvic angiography [27]. Among the 49 pelvic angiography patients, they found 21 patients without IPAL, 19 with multiple IPAL, and nine with a single IPAL. Hagiwara et al. studied a cohort of 234 consecutive PRI patients, 81 of them having had a pelvic angiography with evidence of IPAL in 61 cases [15]. In both studies, pelvic CT scan imaging was not used to assess the presence of IPAL, and angiography was obtained on only a subset of the cohorts. Therefore, a systematic assessment of IPAL incidence was not performed. However, both publications and other ones highlight that terminal branches of the internal iliac artery (especially the superior gluteal, inferior gluteal, obturator, internal pudendal, vesical and lateral sacral arteries) are most often damaged, as they are close to bone and ligamentous structures [15, 16, 23, 26–28]. Rarely, common and external iliac artery lesions may also occur [17, 24, 26]. The present study confirms published data on IPAL associated with high-energy blunt PRI and has the advantage to be a systematic investigation of a cohort of 127 consecutive patients having all had contrast enhanced CT-scan arterial imaging allowing diagnosis and accurate description of IPAL.

The risk of IPAL was significantly higher among patients with anteroposterior and vertical shear and combined mechanism type fractures (Young and Burgess) and also among patients with type C injuries (AO/OTA). A possible explanation for these findings is that these injury patterns might imply higher distracting forces on arteries close to bony and ligamentous structures than less displaced and less unstable lateral compression lesions and type B fractures. Metz et al. reported a higher number of posterior IPAL (i.e. superior gluteal artery) occurring with anteroposterior compression type injuries, a higher number of anterior IPAL (i.e. obturator and internal pudendal arteries) with lateral compression type injuries and no occurrence of IPAL with vertical shear type injuries [27]. Three other papers also reported a higher incidence of IPAL in patients with type C injuries [15, 26, 27]. Although the present study did not find any occurrence of IPAL with type A fractures, which are the ones not classifiable with the Young and Burgess system, this finding must not wrongly reassure the physician in-charge, as previous publications have reported such occurrences [15, 26, 27].

Hemodynamic instability and the need for a higher number of PRBC transfusions were associated with the presence of an IPAL. This seems evident, as any high-pressure arterial source of bleeding might lead to an important amount of blood loss which is practically more difficult to control than venous bleeding [13, 14, 19, 20]. Severe associated extra-pelvic injuries may also have impacted hemodynamic instability and the need for PRBC transfusion in the present study. However, as PRI patients with IPAL did not have more severe extra-pelvic injuries than PRI patients without IPAL, this influence does not seem paramount. Surprisingly, the association of IPAL with hemodynamic instability and higher needs of PRBC did not lead to higher complication or death rates, which were linked to associated severe extra-pelvic injuries. One possible explanation lies in the institutional high-energy PRI management protocol involving early and aggressive use of angiography and selective embolization whenever hemodynamic instability persisted after emergent mechanical stabilization of the pelvis and PRBC transfusion. In the present study, embolization was used in 40% of IPAL patients. This might have led to an early control of arterial bleeding responsible for persisting hemodynamic instability despite PRBC transfusion and pelvic stabilization. The most frequent arterial lesions involved in embolization procedures were on the obturator, superior gluteal, inferior gluteal and vesical arteries. Study design and extracted data did not allow to highlight which specific arterial lesion needed embolization to obtain hemodynamic stability in case of multiple IPAL. However, this topic is beyond the scope of discussion of this paper. Ultimately, hemodynamic instability in case of high-energy blunt PRI should lead to the prompt assessment of the presence of possible IPAL and to their aggressive management with PRBC transfusion and eventual invasive procedures (embolization, REBOA): respective indications for these invasive arterial hemostatic treatment modalities still need to be definitively clarified, and may either involve the treatment of every arterial blush visualized on contrast-enhanced CT-scan even if initial hemodynamic instability was controlled by fluid resuscitation and mechanical pelvic stabilization (assuming that the retroperitoneal cavity lacks any tamponade function), or be performed only by persisting hemodynamic instability, depending on local high-energy trauma patients’ management protocols [14, 18, 19]*.*

The major limitation of this study is the low number of patients with IPAL (15 patients with 21 lesions) which might limit the accuracy of analysis on this subset of patients and lesions. Also, the multivariate analyses have some weaknesses: 1) the low numbers of outcomes may have constrained to select the number of covariates to evaluate; 2) PRI with and without IPAL could not be analyzed as totally isolated injuries, without being influenced by associated extra-pelvic injuries. These weaknesses were probably mitigated by the fact that PRI patients with IPAL did not have more severe extra-pelvic injuries than PRI patients without IPAL. However, this study is the first systematic investigation on a large cohort of consecutive patients with high-energy blunt PRI using a reliable diagnostic tool to highlight IPAL. Therefore, the rate and topography of IPAL found in this study might represent an important point for the aggressive management of high-energy blunt PRI patients.

## Conclusions

This systematic study found an 11.8% rate of IPAL among patients sustaining high-energy blunt PRI. The three most often injured arteries were the obturator artery, followed by the superior gluteal artery and the inferior gluteal artery, which are all branches of the internal iliac artery. The risk of IPAL was significantly higher among patients with anteroposterior and vertical shear and combined mechanism type fractures (Young and Burgess classification) and also among patients with type C injuries (AO/OTA classification). Hemodynamic instability in case of high-energy blunt PRI should lead to the prompt assessment of the presence of possible IPAL and to their aggressive management with PRBC transfusion and eventual invasive arterial hemostatic procedures. This study emphasizes the unique diagnostic role of CT scan with contrast enhanced arterial sequences to point out or rule out arterial bleeding and the need for further therapeutic measures (angiography and embolization, REBOA). These findings are important for the aggressive management of hemodynamic instability in high-energy blunt PRI patients.

## Supplementary Information

**Additional file 1: Supplement Table 1**. Association between intra-pelvic arterial lesions and other extra-pelvic injuries.

## Data Availability

The datasets generated and analyzed during the current study are not publicly available because a formal demand in this sense was not made to the institutional research ethics board at the time of project initiation. However, the above-mentioned datasets are available from the corresponding author on reasonable request.

## Abbreviations

95% CI: 95% confidence interval
AIS: Abbreviated Injury Scale
AO/OTA: Arbeitsgemeinschaft für Osteosynthesefragen / Orthopaedic Trauma Association
CT: computed tomography
DICOM: digital imaging and communications in medicine
IPAL: intra-pelvic arterial lesion
ISS: Injury Severity Score
OR: odds ratio
PACS: picture archiving and communication system
PRBC: packed red blood cells
PRI: pelvic ring injury
REBOA: resuscitative endovascular balloon occlusion of the aorta
ROI: region of interest
SD: standard deviation
VIF: variance inflation factor
