# Supplementary material for: Incidence rate and topography of intra-pelvic arterial lesions associated with high-energy blunt pelvic ring injuries: a retrospective cohort study
Source: BMC Emerg Med. 2021 Jun 30;21:75. doi: 10.1186/s12873-021-00470-y (PMC8243444; doi:10.1186/s12873-021-00470-y)
Supplement: Supplementary file 1 — Additional file 1: Supplement Table 1. Association between intra-pelvic arterial lesions and other extra-pelvic injuries. [file 12873_2021_470_MOESM1_ESM.docx]

**Supplement TABLE 1**: Association between intra-pelvic arterial lesions and other extra-pelvic injuries

|  | **Arterial lesion** | |  |
| --- | --- | --- | --- |
| **Classification** | **Absent (n=112)** | **Present (n=15)** | **p-value** |
| **AIS head & neck** |  |  | 0.264^1^ |
| ≤2 (n=84) | 76 (67.9) | 8 (53.3) |  |
| >2 (n=43) | 36 (32.1) | 7 (46.7) |  |
| **AIS thorax** |  |  | 0.338^1^ |
| ≤2 (n=57) | 52 (46.4) | 5 (33.3) |  |
| >2 (n=70) | 60 (53.6) | 10 (66.7) |  |
| **AIS abdomen** |  |  | 0.319^2^ |
| ≤2 (n=99) | 89 (79.5) | 10 (66.7) |  |
| >2 (n=28) | 23 (20.5) | 5 (33.3) |  |

Values are expressed as n (%).

^1^ Chi-2 test.

^2^ Fischer's exact test.

*AIS:* Abbreviated Injury Scale. An AIS ≤2 represents a minor or moderate injury, whereas an AIS >2 represents a serious, severe or critical injury.
